# Prediction and analysis of near-road concentrations using a reduced-form emission/dispersion model

**DOI:** 10.1186/1476-069X-9-29

**Published:** 2010-06-25

**Authors:** Stuart A Batterman, Kai Zhang, Robert Kononowech

**Affiliations:** 1 Department of Environmental Health Sciences, University of Michigan, Ann Arbor, MI 48109, USA

## Abstract

**Background:**

Near-road exposures of traffic-related air pollutants have been receiving increased attention due to evidence linking emissions from high-traffic roadways to adverse health outcomes. To date, most epidemiological and risk analyses have utilized simple but crude exposure indicators, most typically proximity measures, such as the distance between freeways and residences, to represent air quality impacts from traffic. This paper derives and analyzes a simplified microscale simulation model designed to predict short- (hourly) to long-term (annual average) pollutant concentrations near roads. Sensitivity analyses and case studies are used to highlight issues in predicting near-road exposures.

**Methods:**

Process-based simulation models using a computationally efficient reduced-form response surface structure and a minimum number of inputs integrate the major determinants of air pollution exposures: traffic volume and vehicle emissions, meteorology, and receptor location. We identify the most influential variables and then derive a set of multiplicative submodels that match predictions from "parent" models MOBILE6.2 and CALINE4. The assembled model is applied to two case studies in the Detroit, Michigan area. The first predicts carbon monoxide (CO) concentrations at a monitoring site near a freeway. The second predicts CO and PM_2.5 _concentrations in a dense receptor grid over a 1 km^2 ^area around the intersection of two major roads. We analyze the spatial and temporal patterns of pollutant concentration predictions.

**Results:**

Predicted CO concentrations showed reasonable agreement with annual average and 24-hour measurements, e.g., 59% of the 24-hr predictions were within a factor of two of observations in the warmer months when CO emissions are more consistent. The highest concentrations of both CO and PM_2.5 _were predicted to occur near intersections and downwind of major roads during periods of unfavorable meteorology (e.g., low wind speeds) and high emissions (e.g., weekday rush hour). The spatial and temporal variation among predicted concentrations was significant, and resulted in unusual distributional and correlation characteristics, including strong negative correlation for receptors on opposite sides of a road and the highest short-term concentrations on the "upwind" side of the road.

**Conclusions:**

The case study findings can likely be generalized to many other locations, and they have important implications for epidemiological and other studies. The reduced-form model is intended for exposure assessment, risk assessment, epidemiological, geographical information systems, and other applications.

## Background

The use of geocoded data and geographical information systems (GIS) has rapidly becoming routine practice in many types of environmental analyses, including risk assessment and environmental epidemiology. Most studies have used surrogates of pollutant exposure, including proximity measures such as the distance from residences or schools to highways or Superfund sites. While easy to display and analyze within a GIS, proximity is at best a crude surrogate of exposure since it incompletely accounts for the nature of emission sources, effects of meteorology, orographic features and other factors that affect pollutant emissions, transport, fate and exposure. Further, quantitative exposure estimates are not obtained [1]. Relatively few studies have used emission and dispersion models to predict exposures to ambient air pollutants. Such models, which can predict spatially- and temporally-resolved concentrations, have the potential to improve exposure estimates and facilitate new types of analyses.

Approaches for estimating air pollutant exposures from roadways have been reviewed by Lipfert and Wyzga [2] and HEI [3]. As mentioned, most studies have used proximity as a surrogate of exposure, most often the distance between the subject's residence and highway, although several studies have used other measures, e.g., traffic intensity [4]. While quite easy to derive within GIS framework, a significant drawback of proximity and traffic intensity measures is the potential for biased and misclassified exposure estimates since such measures do not consider effects of meteorology, vehicle emissions, and time-activity patterns of the study subjects, e.g., time spent away from the location considered. Moreover, such measures are unlikely to properly account for the small scale variation in pollutant concentrations [1].

Simulation models have been used to evaluate near-roadway impacts of traffic-related air pollution in a variety of applications [5-10]. These models utilize emission and dispersion components, the latter typically based on the Gaussian plume equation. Such models can be data-intensive, requiring data on pollutant emissions, emission source and roadway configurations, meteorological conditions, and land use parameters. CALINE4 is one of the more popular Gaussian-based line source models [11]. With appropriate input data, simulation models can be used to predict short- and long-term air pollution concentrations at desired locations called "receptors," and multiple receptors can be used to represent spatial and temporal gradients at regional, urban and local scales. The development of the site-specific emission information that "drives" such models is not trivial. Vehicle emissions depend on many factors, including the number, speed, type and age of vehicles, all of which can vary significantly over the course of a day. Emission/dispersion models do not require data from existing pollutant monitoring sites to estimate near-road concentrations and exposures, although such information may be used to estimate the "background" component of concentrations contributed by other "local" and "regional" emission sources, i.e., those not explicitly modeled because they are distant, too numerous, or too difficult to simulate. The drawbacks of dispersion models include, among others, extensive input data requirements, errors due to unmeasured variability in emissions and other parameters, the need for accurate locational information, simplified and possibly unrealistic model assumptions; the relevance of the background estimates, and a need for validation.

Another type of process-based modeling uses computational fluid dynamic (CFD) models [12]. Based on the Navier-Stokes equations, such models are useful for estimating short-term dispersion of plumes, especially in areas containing obstacles like large buildings and complex terrain, and with calm or very light winds, a situation when other types of models perform poorly. However, CFD models are especially demanding in terms of data inputs and computational requirements, and they are not immune to many of the other drawbacks just discussed for dispersion models.

A fourth and recent approach for estimating air pollutant exposures, called "land use regression" (LUR) models, fit concentrations measured at multiple sites using statistical models and land characteristics, traffic and other data as independent variables, which then are used to predict pollutant concentrations at other sites [13]. The primary advantage of LUR models is their ability to characterize small-scale variations in urban settings without the need for detailed (and accurate) emission information. However, these models are area-specific and cannot be reliably extrapolated to areas with different topography, land uses, emission types, etc. Since monitored pollutant levels are used as the dependent variable in the regression model, they also require a network of air sampling sites and historical data. LUR models have been used to estimate only long-term concentrations.

This paper focuses on near-road exposures of traffic-related air pollutants, which have been receiving increased attention due to evidence linking emissions from high-traffic roadways to asthma aggravation, impaired lung function, increased cardiovascular mortality, increased all-cause mortality, and other adverse health effects [3,14,15]. The development of relatively simple, reliable and "user-friendly" models that can be easily linked to a GIS or used in other analyses would benefit researchers needing to generate reliable predictions of pollution concentrations.

The first objective of this paper is to develop a streamlined model for microscale analyses, specifically, to predict short- and long-term air pollution concentrations of carbon monoxide (CO), particulate matter below 2.5 μm dia (PM_2.5_) and other air pollutants near roads that match widely-used and validated emission and dispersion models. The reduced-form model has several advantages over existing models, including the ability to predict concentrations for an arbitrarily large number of receptors and time periods, fast computations, and relatively limited data needs. All of this can facilitate use of the model in exposure assessment, epidemiology and risk assessment applications, especially if predictions are needed for a large number of receptors and/or road segments. Also, the simple form of the reduced-form model permits easily incorporation into GIS and other applications. The second objective of this paper is to identify critical variables, exposure patterns and knowledge gaps that should be recognized in exposure and risk assessment applications addressing near-road exposures.

The paper is organized as follows. We first review approaches for estimating exposures from vehicles. The development of reduced-form submodels to simulate emissions and dispersion is then described. This involves the use of response surface techniques for key variables, which are assembled in a modular fashion to facilitate development and verification. Sensitivity analyses identify critical variables and illustrate the model's behavior. We then select key variables and derive parameterizations for the reduced-form model. The assembled model is demonstrated using two case studies. The first compares predictions of CO to concentrations monitored near a major freeway. The second highlights issues in exposure assessment by predicting CO and PM_2.5 _concentrations in an area surrounding a major freeway and an arterial road. These applications show several surprising and important results regarding the distribution, spatial and temporal variability of concentration predictions. We close on comments regarding implications for exposure and risk assessment, and limitations of the model.

## Methods

### Emission modeling

The first of two submodels, which predicts hourly estimates of vehicle emission rates, is based on MOBILE6.2 model, a macroscopic model developed by the U.S. Environmental Protection Agency that is widely used in emission inventory and dispersion modeling applications. Model predictions are based on emission tests using standard driving cycles designed to represent typical driving patterns along different types of roads, e.g., freeway, arterial, ramp and local roads [16]. Our goals were to match MOBILE6.2 predictions given the information that is typically available for mobile source modeling, to incorporate the major factors that affect vehicle emissions, and to strike a reasonable balance between simplicity and the ability to predict emissions. We were primarily concerned with exposures near large roads, i.e., freeways and arterials, and did not examine idling, cold start, and other types of emissions. This task was facilitated by previous sensitivity analyses that have identified critical model inputs, including those using conditions relevant to the Detroit case study [9,17]. After selecting critical inputs, we used a set of lookup tables for emission factors organized by year and pollutant, taken directly from MOBILE6.2 outputs, and then estimated emissions on the segment by scaling up the emission factors by the road's vehicle mix and volume.

### Dispersion modeling

The second submodel was developed to represent CALINE4, a line-source Gaussian plume dispersion model originally developed by the California Department of Transportation to predict 1- and 8-hr CO concentrations at pre-determined receptor positions near roadways [11]. The model can also simulate formation and dispersion of NO_2_, using a simple set of reactions to predict its formation from precursors NO and O_3_, and PM, using algorithms to model deposition and settling processes. Required inputs include roadway geometry, hourly surface meteorology, traffic volume and emission rates. Individual highway segments are divided into a series of elements, each modeled as an "equivalent" finite line source that is normal to the wind direction and centered at the element's midpoint, from which incremental concentrations are computed and summed to predict the concentrations at designated receptors. We derived a reduced-form dispersion submodel using multiplicative parametric equations that are simple to implement and solve, essentially representing a response surface analysis for individual processes in the model.

**Figure 1:**
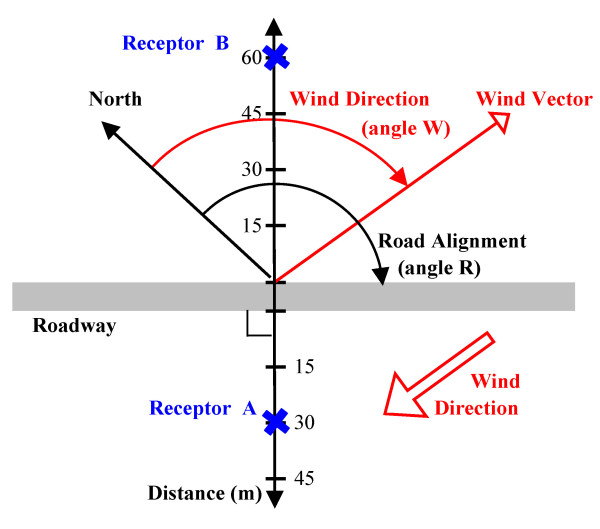
**Depiction of road and receptor coordinate system for arbitrary road and wind directions**.

The CALINE4 documentation includes sensitivity analyses for selected model inputs [11]. To guide the development of the reduced-form model, we performed sensitivity analyses for key parameters that were individually varied over a wider range than analyzed previously, while other parameters were maintained at nominal values. Figure 1 defines several of the parameters used in this analysis. Road alignment angle R is defined from north with a range of 0 to 180° (e.g., 90° = east-west alignment). Receptors are defined with respect to the road by distance x (m) measured normal from the road centerline. Wind angle θ is defined for a given receptor such that θ = 0° indicates that the wind is perpendicular to the road but that the receptor is upwind of the road; θ = 90° or 270° are winds parallel to the road; and θ = 180° is again for a wind perpendicular to the road, but this time the receptor is downwind.

Most of CALINE4 analyses used 1 hr runs, a straight roadway element 2 km in length, flat surrounding terrain, a set of receptors aligned normal to the road at the segment's center, downwind distances from 15 to 300 m at 15 m intervals, and a receptor height of 1.8 m. The nominal case also assumed: mixing height = 500 m; ambient temperature = 15°C; background concentration = 0 ppm; atmospheric stability category (SC) = D (the most common case); wind speed = 4 m s^-1^; vehicle volume = 10,000 vehicles hr^-1^; road at grade level; mixing zone width = 30 m, and an artificially high emission rate to obtain sufficient precision in model outputs. Model predictions were subsequently adjusted to derive concentrations for a nominal emission rate of 1 g km^-1^. The sensitivity analysis varied the following inputs: each SC A through F (for this, we used a wind speed of 2 m s^-1 ^since SC F is not defined at 4 m s^-1^); wind speeds of 1, 2, 4, 6, 8, 10 and 12 m s^-1^; wind angles from 0 to 180° in 10° increments, sufficient given the symmetry of the problem; vehicle volumes from 1,000 to 15,000 vehicles hr^-1 ^in increments of 2,000; the road at grade, 6 m below grade, and as a bridge 6 m above grade; mixing heights of 25, 50, 100, 200, 500 and 1000 m; and mixing zone widths of 20, 30 and 45 m. Predicted concentrations at each downwind distance were graphically displayed. Previous sensitivity analyses have examined wind direction variability, surface roughness, deposition velocity, highway geometry (including width, height, length), and other factors [11]. These can be important in special cases, but they generally represent secondary influences.

### Reduced-form dispersion model

A reduced-form dispersion model using analytical expressions was derived that obtained comparable predictions to CALINE4, guided by the results of the sensitivity analysis. A multiplicative and modular model structure using sub-models for each major input parameter was selected, thus allowing easy updates. We attempted to strike a balance between reproducing CALINE4's output exactly, using readily available data, and keeping calculations fast and simple, and we maintained those inputs that changed predicted concentrations by more than 10 or 15%. This criterion applies only to the nominal conditions modeled, e.g., flat terrain, and road at grade level. Generally, input parameters making smaller differences were omitted. A variety of model structures for the submodels were evaluated, including exponential, power law and polynomial regression models, among others, and parameter coefficients were estimated using maximum likelihood estimates and non-linear Newton gradient search procedures.

Like other Gaussian dispersion models, calm winds cannot be accurately modeled. We set the minimum wind speed to 0.5 m s^-1^. For calms, no calculations were attempted (the hour's concentration was recorded as not available). Daily averages were calculated if at least half of the hourly observations were available.

### Model evaluation

We first verified the reduced-form model by examining inter-model agreement using correlations, relative errors, absolute relative error statistics, and scatter plots. The performance of the reduced-form model was evaluated over the full range of input parameters. Next, we conducted a limited evaluation of the reduced-form model by comparing hourly and daily average predictions to CO measurements for the year 2004 at the Allen Park, Michigan monitoring site, which is operated by the Michigan Department of Environmental Quality (MDEQ). The site was selected due to its proximity to both a major freeway and a permanent traffic recorder (PTR), which records hourly traffic volume. The site is 17 km SW of downtown Detroit in a flat and largely tree-free area, just north of Goddard Road and 150 m SE of interstate I75, which oriented at 40° (although it curves just S of the site). The surrounding land use is primarily residential, although there are various commercial and industrial facilities within 5 km of the site. CO is monitored using U.S. EPA approved instrumentation (DASIBI 3008 analyzer). Surface meteorological observations are also collected at this site. For 2004, the annual average daily traffic (AADT) near the monitoring site was 101,000 vehicles day^-1^, and the commercial average daily traffic (CADT) was 13,500 vehicles day^-1^. (These values are lowered by 6% from the PTR measurements to account for egress of vehicles prior to the Allen Park location, which is 3.5 km from the PTR [18]). We obtained hourly CO, meteorological, and PTR data for 2004. Most (> 95%) of the CO and meteorological data were available; traffic count data had lower availability (74%). After row-wise eliminations (including calms), 6,046 hours, representing 263 days with most data available, were available. We predicted CO hourly and daily CO concentrations using the reduced-form model, the monitored traffic flow, the regional 2004 vehicle age distribution, the local vehicle mix [17], and the Allen Park meteorological data. Due to some local features that appeared to influence wind direction, primarily a line of trees 25 m N, we used hourly wind direction data from the local airport, located 18 km to the west of the monitoring site.

### Detroit case study

To demonstrate a more complex application, we modeled a 1 km^2 ^area of Detroit, Michigan around the intersection of a freeway (M39, the Southfield Expressway) and arterial road (M5, Grand River). We set up a rectangular receptor grid consisting of 43 rows by 41 columns on 25 m centers (1935 receptors; Additional file 1: figure S1). Hourly traffic volume, speed and emission rates were estimated for the two roads and each vehicle class used in MOBILE6.2, based on weekday road-specific volume measurements, and the regional traffic mix (which distinguishes road type [19]. We adjusted the hourly volumes for Saturday, Sunday and holiday periods using factors derived from the hourly year-round PTR measurements on I-75 (described previously) since traffic volume measurements for the two roads were available for only weekday periods. Hourly meteorological data was obtained from the local airport, located 23 km SW of the study region. We then used the reduced-form model to predict CO and PM_2.5 _concentrations at each receptor for each hour in 2004, which were processed into daily averages. Annual average and selected percentile concentrations were examined and plotted, as were "best case" and "worst case" conditions, selected on the basis of the lowest and highest concentration averaged across the modeled domain.

## Results

### Sensitivity analysis of MOBILE6.2

**Figure 2:**
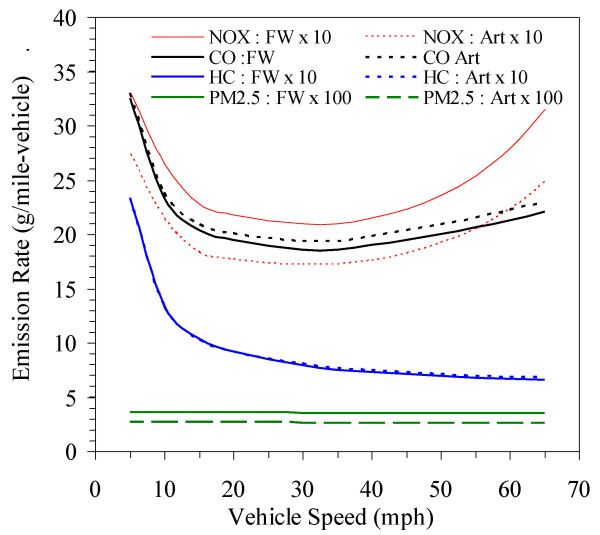
**Estimated composite vehicle emission rates as function of speed, averaged over vehicle mix in the case study on both freeways (FW) and arterial roads (ART)**. Uses winter emission rates.

Results from previous sensitivity analyses have shown that MOBILE6.2 emissions are most sensitive to several inputs [11,20]:

• Vehicle speed is a strong predictor. Figure 2 shows vehicle emissions as a function of speed using highway and arterial vehicle mixes in the Detroit case study and winter temperatures. CO and NO_x _emissions show the classical "U-shaped" emission curve; PM_2.5 _emissions are unaffected by vehicle speed. Road type influenced CO and NO_x _(discussed below). Light duty gas vehicles (LDGVs), which represented 45 - 48% of the total traffic volume (and more on arterials) emitted 59% of the CO, 20 - 26% of the NO_x _(more on arterials) and 56% of the HC. Heavy duty diesel vehicles (HDDVs), which represented 7 - 11% of the traffic (more on freeways), account for 45 - 55% of the NO_x _(more on freeways), and 69 - 75% of the PM_2.5 _(more on freeways). The other vehicle classes contributed the remainder of emissions, particularly light duty gas trucks.

• Vehicle type is important, e.g., HC and CO emissions are highest for heavy duty gasoline vehicles (HDGVs), while HDDVs dominate NO_x _emissions and PM_2.5 _emissions.

• Vehicle age distribution, which differs by region and year modeled, is a key predictor. In comparison to the U.S. average, the Detroit area age distribution (newer than the national average) gave 2010 emission rates that were lower than the national average by 41% for VOCs in summer; 18% for NO_x _in summer, and 27% for CO in winter [17]. Comparing annual averages from 2004 to 2006, CO emission rates dropped by 15% (freeways) to 19% (arterials), and by 20% for PM_2.5_. These effects are caused by the retirement of older and more polluting vehicles.

• Road type shows limited sensitivity, particularly for newer vehicles. Emission rates for freeways, arterials, ramps and local roads for the 2010 vehicle age distribution were similar for all pollutants except CO, where ramps increase emissions by about 15%, and local roads, where CO emissions are about 10% lower, as compared to freeways and arterial roads. (Vehicle mix varies by road type.)

• Ambient temperature's effect depends on the pollutant. VOC emissions are lowest at about 20°C and increase 15-20% in both cold and warm temperatures. With increasing temperatures, NO_x _decreases by about 20% and CO decreases by nearly a factor of two [17]. PM_2.5 _emissions are insensitive to temperature.

• Fuel parameters are important. Sulfur in fuel strongly affects SO_2 _and PM emission rates. From 2000 to 2010, the default assumptions in MOBILE6.2 for sulfur content in gasoline dropped from 300 to 30 ppm; for diesel, the model does not include default assumptions but levels have also dropped considerably, e.g., typical sulfur content in diesel fuels for 1995-2005 was ~500 ppm, dropping to 15 ppm beyond June 2006 due to the heavy-duty diesel rule requirements. Other fuel parameters can also be important, e.g., Reid vapor pressure strongly affects evaporative (but not tailpipe) emissions of VOCs.

The model is generally insensitive to relative humidity, trip length, and the number of starts per day (for on-road emissions).

### Reduced-form sub-model for road segment emissions

Based on the available information and sensitivity analyses, we decided to maintain information regarding vehicle speed, vehicle age distribution, vehicle type, traffic volume, ambient temperature, and fuel sulfur content in the reduced-form model. We used constant and default values for other parameters, including road type and relative humidity, to which the model demonstrated very limited sensitivity.

Information regarding traffic volume, vehicle speed, age distribution and vehicle mix on specific road segments is generally is limited. In the case study area, for example, available information included: hourly measurements of traffic volume at multiple locations on the major roads for a few weekdays; estimates of vehicle age distributions across the Detroit area; estimates of hourly vehicle mix on arterial and freeways, also across the Detroit area; posted speed limits; and speed estimates for four periods per weekday. We estimated travel speeds on an hourly basis using the Bureau of Public Road (BPR) method, one of the more popular approaches [21]. The mean travel speed S_I, T _(mph) for on a road segment or "segment I and hour T is:(1)

where S_I, FF _= free flow speed limit on road segment I (mph); V_I, T _= hourly traffic volume on the segment at hour T (vehicles hr^-1^); C_I _= road capacity for the segment (vehicles hr^-1^), estimated locally as 2000 vehicles hr^-1 ^lane^-1 ^for freeways and 825 vehicles hr^-1 ^lane^-1 ^for urban arterials;[19] α = coefficient ranging from 0.05 to 1; and β = power coefficient ranging from 4 to 11. Various α and β have been used, and values are often calibrated to reflect local conditions. We estimated coefficients for the case study using speeds and volumes measured during several morning and evening periods, giving α = 0.1226 for the freeway, α = 1.00 for the arterial, and β = 4.688. Generally, eq. 1 yields only small decreases in speeds for roads below capacity, e.g., at capacity, freeway speeds drop from 60 to 53.4 mph, and urban arterial speeds drops from 35 to 17.5 mph. In the case study, capacity was never reached, and speeds never decreased by more than 5 mph from free flow speeds, even at peak volumes.

The emission rate on weekdays for segment I at time T, Q_I, T _(g h^-1 ^km^-1^), is determined as:(2)

where 0.625 = conversion to km from mi; V_D, I, T _= total traffic flow for segment I, day D, and hour T (vehicles hr^-1^); M_D, I, K, T _= mix (fraction) of vehicles for vehicle class K on segment I, day D and time T; Q_D, K, T, SD, I.T _= emission rate (g mile^-1 ^vehicle^-1^) for day D, vehicle class K, time T and segment speed S_D, I, T _(mph); and S_D, I, T _= segment speed estimated using eq. 1. Q_D, K, T, SD, I.T _was estimated using MOBILE6.2, the assumed vehicle age distribution within the class (g mile^-1 ^vehicle^-1^), [19] and eight vehicle classes (LDVs, light duty truck 1, light duty truck 2, light duty truck 3, light duty truck 4, heavy duty truck, heavy duty bus, and motorcycle). Since MOBILE6.2 predictions depend only weakly on ambient temperature, we simplified the derivation of Q_D, K, T, SI, T _by estimating emissions seasonally (rather than hourly) using ambient temperatures of 28.8, 51.1 and 72.3°C for winter, spring/fall and summer seasons, respectively [19]. We predicted emissions every 5 mph from 5 to 65 mph in each season, and used a lookup table to match the predictions closest to the segment's estimated speed.

Hourly estimates of V_D, I, T _and M_D, I, K, T _are available for typical weekday periods, but generally not for each hour of the year. We assume that the available pattern holds for non-holiday weekdays throughout the entire year. We developed three additional patterns to represent Saturdays, Sundays and major U.S. holidays (New Years Day, Memorial Day, Independence Day, Labor Day, Thanksgiving, Christmas). The total hourly traffic volume (across all vehicle classes) for weekdays, weekends and holidays is:(3)

where V_D, I, T _= total volume for day type D (weekday, Saturday, Sunday and holiday), segment I, and hour T (vehicles hr^-1^); V_WD, I, T _= total volume on typical weekdays on segment I at hour T; and F1_D, T _= adjustment factor for day type D and hour T. (F1_D, T _for weekdays = 1.) F1_D, T _was calculated using hourly 2004 traffic counts from the I-75 PTR (described previously), and values ranged from 0.21 (early Sunday 8 am) to 1.37 (Saturday 1 am; Table S1). To obtain hourly volumes for each vehicle class (needed to estimate M_D, I, K, T _= V_D, I, K, T _/V_D, I, T_), we used local data as follows. First, the unadjusted volume was estimated as:(4)

where V*_D, I, K, T _= unadjusted volume for day type D, segment I, vehicle class K, and hour T (vehicles hr^-1^); V_D, I, T _= total volume from eq. 3; F2_WD, I, K, T _= weekday vehicle mix factor (dimensionless), representing the fraction for segment I, vehicle type K and hour T; and F3_D, H, RT, VC _= ratio (dimensionless) of traffic volume for day type D, segment I, vehicle class K, and hour T to the weekday volumes for the same I, K, and T. F2_WD, I, K, T _is available for typical weekdays and typical road types (supplemental Table S2). F3_D, I, K, T _was estimated from the PTR data for Saturday, Sunday and holiday periods for light and heavy duty vehicles (separately), and was applied to both freeway and arterial roads. Lastly, results were normalized to obtain the correct daily volume:(5)

The effect of these adjustments is shown in Additional file 1: figures S2 - S5). As examples: on weekends and holidays, volumes are reduced overall and the morning rush hour peak is eliminated; heavy duty-vehicles show different patterns than the total volume, which is dominated by LDVs; and truck volumes on non-weekday evenings are particularly low. We also compared the approach represented by eqs. (3-5) to the 13 classifications, mainly based on weight, given by the PTR.

### Sensitivity analysis of CALINE4

**Figure 3:**
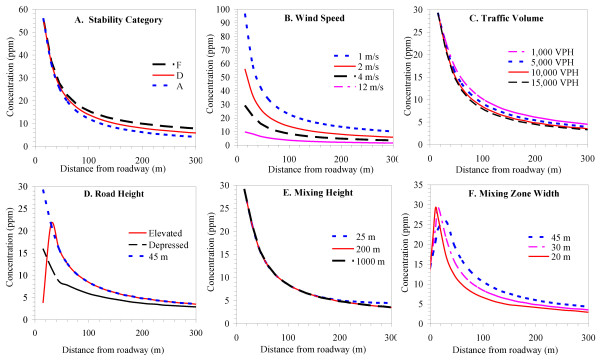
**Predicted CO concentrations showing sensitivity to: A) atmospheric stability category; B) wind speed; C) mixing height; and traffic volume**. Unless otherwise modified, plots use nominal conditions (wind speed = 4 m s^-1^, VPH = 10,000; emission rate = 300 g mi^-1^; wind angle = 180°). Panel A uses a wind speed of 2 m s^-1^. Panel C uses a constant link emission rate.

Figure 3 depicts results of the sensitivity analysis, in which individual parameters were varied from the nominal condition. For stability category (SC), the largest effect is seen at long distances from the roadway (Figure 3A). For example, in comparison to concentrations predicted under SC F (most stable giving the highest concentrations, concentrations under SC A (most unstable) were 8% lower at a distance of 50 m, 23% lower at 100 m, 31% at 150 m, and 47% lower at 300 m. Changing the SC from A to F is the most extreme comparison possible, and in many areas, these two SCs are uncommon (occurring less than 5% of the time). Predictions for the more common SCs were similar, e.g., changes from F to B ranged were within 1 to 12% for the same comparisons just discussed. We also note that the greatest differences occurred at relatively large distances when roadway impacts are not likely to be large. For these reasons, we conclude that SC has only moderate influence on CALINE4 predictions. This conclusion differs from point source modeling in which stability category is one of the most sensitive parameters. Line source models for vehicles are much less sensitive since the amount of initial mixing induced by mechanical and thermal turbulence is relatively large, which reduces the importance of ambient stability near the roadway [11]. Additionally, crosswind (horizontal) dispersion parameters have small effects in line source models, and both sources and receptors are near ground level and thus always "in" the plume. Greater sensitivity to SC can result in some cases, e.g., low traffic volumes when vehicle-induced turbulence is less significant [11].

**Figure 4:**
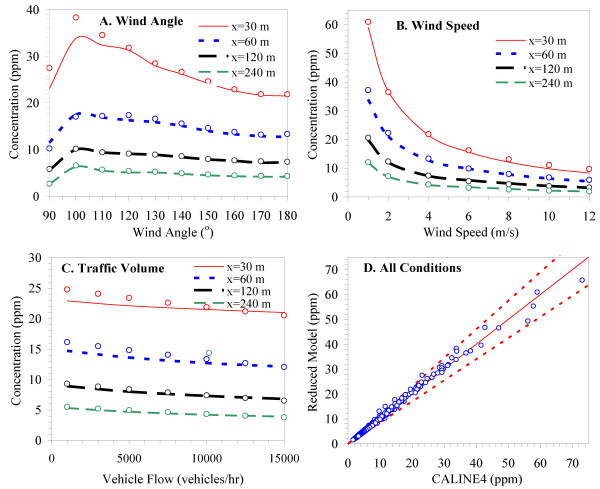
**Comparison of CALINE model predictions (shown as solid lines) and reduced model predictions (shown as points) showing effects of A) wind angle; B) wind speed; and C) traffic volume for four distances (30, 60, 120, 240 m) from the road**. All plots use nominal conditions (wind speed = 4 m s^-1^, VPH = 10,000; emission rate = 300 g mi^-1^; wind angle = 180°). Panel D plots concentrations from the two models for all conditions showing 1:1 line and 15% error intervals.

CALINE4 predictions strongly depend on wind angle, and the highest concentrations outside the mixing zone are produced by a wind angle of ~10° as measured from the road centerline; the highest concentrations on the roadway occur for winds parallel to the road [11]. (Results shown later in Figure 4A.)

Wind speed had a strong effect, e.g., predictions dropped by 80% as winds increased from 1 to 10 m s^-1 ^(Figure 3B). Wind dilutes pollutants in an inverse manner, but the initial vertical and horizontal dispersion parameters also depend on wind speed, which slightly weakens the dilution effect [11].

Higher traffic volume increases CALINE4 predictions due to its proportional relationship with emission rates. However, the effect on concentrations is less than proportional since higher volumes also increase dilution due to vehicle-induced heat fluxes that increase vertical dispersion. The countervailing effect is stronger for winds parallel to the road, and diminished for crosswind conditions at locations outside of the mixing zone [11]. For the purpose of the sensitivity analysis, we held the emission rates constant when changing traffic volume. For example, a 10-fold increase in traffic (1,000 to 10,000 vehicles hr^-1^) with winds perpendicular to the road (wind angle = 180°; the nominal case in the sensitivity analysis), concentrations at the curbside did not change, but decreased by 17% at a distance of 100 m and 22% at 300 m (Figure 3C). For winds nearly parallel to the road (wind angle = 100°), the same change decreased concentrations by 13% at the curbside and by 29% for distances from 90 to 300 m.

Concentrations were sensitive to road height, particularly near-road receptors (Figure 3D). When the roadway was depressed below grade, concentrations decreased by 45% close to the road; the decrease was smaller (17%) at long downwind distances. Depressed roadways are simulated by increasing the residence time in the mixing zone, which increases vertical mixing and lowers concentrations. For elevated roads/bridges, concentrations are low at receptors very near the road, but at longer distances, concentrations are identical to those attained for roads at grade. This pattern results as CALINE4 assumes uninterrupted wind flows beneath the bridge, thus elevating the plume over near-road receptors. The size of these effects depends on the vertical distance above or below grade level.

Mixing height had virtually no effect on predicted concentration (Figure 3E). Vertical dispersion in the microscale region is small relative to normal mixing height of 100 m or more, although sensitivity to mixing height increases under unstable atmospheric conditions [11].

CALINE4 defines the mixing zone width as the roadway width plus 3 m on both sides. Wider roads increase the residence time and the initial horizontal distribution of the source, enhancing both vertical and horizontal dispersion [11]. However, this effect is more than offset by the closer proximity of the mixing zone to the receptor. Thus, wider mixing zones are associated with higher concentrations (Figure 3F). For example, increasing the mixing zone width from 30 to 45 m increased concentrations by 24%.

The CALINE4 sensitivity analysis was conducted using CO, for which deposition and settling processes have negligible effect [11]. However, these results should also apply to PM_2.5 _emissions from vehicle exhaust, which form very small particles (well below 1 μm in dia), since these processes also will have only minor effects, at least at the short distances considered [22]. Additional processes sometimes relevant in modeling PM concentrations include coagulation for the ultrafine fraction, precipitation scavenging, entrainment of roadway dust, and PM emissions from tire and brake wear.

### Reduced-form dispersion submodel

We developed a reduced-model form of CALINE4 with variable selection and structure based on the sensitivity and additional analyses. Of the factors evaluated, we deemed that mixing height and stability category had only minor effects in most cases and did not warrant inclusion. For simplicity, because information regarding the roadway height above or below grade for complex road networks is generally unavailable in GIS shape files, and because effects were not large past about 100 m, we assumed that roads were at grade level. For similar reasons, we did not account for the mixing zone width, and assumed a width of 30 m, which suits many larger roads. We modeled the remaining parameters using multiplicative submodels.

One of the more complex submodels fits concentration profiles from the road for each wind angle and downwind distance, parameters that had large impacts on predictions. After testing a number of expressions, we found that a double exponential closely matched the concentration profile seen for distances from 15 to 300 m for each wind angle. For a given wind angle, wind speed, SC, traffic flow and vehicle mix, concentrations could be predicted by:(6)

where C_X _= predicted concentration (ppm) at distance X (m); k_1_, k_2 _and k_3 _= fitted coefficients representing the scale, off-set and decay for the first exponential decay; and k_4_, k_5 _and k_6 _= similar coefficients for the second exponential decay. The two exponential terms represent fast and slow decay processes. Typically, the fast component was stronger and dropped off with a half-distance x_1/2 _(distance for concentration to drop to 1/2 of the initial concentration) of 5 to 15 m, depending on the wind angle. The slow component had a smaller effect and half-distances from 30 to 150 m. Parameters k_1 _to k_6_, shown in Table 1, were estimated for 18 wind sectors (every 10° for wind angles from 0 to 170°). The problem is symmetrical, e.g., a wind angle of 10° is equivalent to 350°. Eq. 6 using the estimated parameters matched CALINE4 predictions within 3% in most cases. The largest differences (errors from 3 to 17%) occurred with wind angles of 90 to 100° and distances from 15 to 75 m.

**Table 1:** Estimated parameters that depend on wind angle for reduced-form CO model giving results in ppm


**Wind Angle (°)**		**Parameter**					
		
		k_1_*	**k_2_**	**k_3_**	k_4_*	**k_5_**	**k_6_**

0	360	0.00000	0.00000	0.000000	0.00000	0.00000	0.00000
10	350	0.00000	0.00000	0.000000	0.00000	0.00000	0.00000
20	340	0.00000	0.00000	0.000000	0.00000	0.00000	0.00000
30	330	0.00150	15.00000	1.000000	0.00000	0.00000	0.00000
40	320	0.00450	15.00000	1.000000	0.00000	0.00000	0.00000
50	310	0.00749	15.00000	1.000000	0.00000	0.00000	0.00000
60	300	0.01049	15.00000	1.000000	0.00000	0.00000	0.00000
70	290	0.01798	15.00000	1.000000	0.00000	0.00000	0.00000
80	280	0.04796	15.00000	1.000000	0.00000	0.00000	0.00000
90	270	0.17290	9.97114	0.006433	1.01566	12.00727	0.07714
100	260	0.21985	1.67889	0.003459	0.83589	14.95353	0.05500
110	250	0.20692	0.12304	0.003792	1.08186	2.38274	0.04362
120	240	0.18484	0.10808	0.003542	0.85176	0.64258	0.03523
130	230	0.17297	0.21253	0.003417	0.67201	0.25399	0.03149
140	220	0.18214	0.34664	0.003888	0.60689	1.05519	0.03347
150	210	0.15555	0.00000	0.003452	0.56272	0.00000	0.03094
160	200	0.15754	0.00000	0.003679	0.50882	0.11021	0.03178
170	190	0.15570	0.10760	0.003731	0.45450	1.95509	0.03241
180	180	0.14206	0.94140	0.003408	0.47168	0.00000	0.02969


Based on eq. (5) in text. Additional parameters are k_8 _= -0.73912; k_9 _= 0.99898; k_10 _= -0.000026515; k_11 _=10749; k_12 _= -0.027061; and k_13 _= 5.2826. Both wind angles shown use the listed parameters. Applies to an emission rate of 1 g km^-1 ^hr^-1^.

A means to estimate wind angles for all possible road and receptor geometries is needed. Referring to Figure 1, this was accomplished by considering road alignment angle R, road-receptor distance x (m), wind angle θ, and a new variable I_W_, which is an indicator variable set to 1 if the receptor is west of the road, and otherwise 0. The wind angle is obtained as:(7)

where MOD_1 _= modulus function with a divisor of 1; W = wind direction (°); I_W _= indicator variable for west direction; and I_R _= another indicator variable set to 1 if the road alignment angle R exceeds 90° (I_R _= 0 otherwise). As an example, Figure 1 depicts wind direction W = 112.5° (ESE) and road alignment angle R = 135° (SE-NW), for which θ = 247.5° for downwind receptor A, and θ = 67.5° for upwind receptor B. Winds from 45 and 225° bring winds perpendicularly across the road to receptors A and B, respectively (giving θ = 180° in both cases).

Wind speed had an approximately inverse power law relationship with concentrations(8)

where C_U _= concentration (ppm) at wind speed U (m s^-1^), k_7 _and k_8 _= estimated parameters, and C_U = 4 _= concentration predicted at the nominal wind speed of 4 m s^-1^. Parameters k_7 _and k_8 _were estimated as 2.81 and -0.739, respectively, over the wind speeds (1 - 12 m s^-1^) and distances (15 - 300 m) considered. This model had an average absolute error of 3.3% for wind speeds and distances from 45 to 300 m; very short distances (15 to 30 m) had larger errors, e.g., predictions from 15% lower (for winds of 1 m s^-1^) to 35% higher (12 m s^-1^). These errors occurred at only the shortest distances.

As mentioned, higher vehicle traffic increases vertical dispersion, and the effect on concentration depends on wind angle and downwind distance. We tried a number of model forms and parameterizations, and attempted to fit winds that were both perpendicular and parallel to the road. The selected submodel is:(9)

where C_V, X _= concentration at vehicle volume V (vehicles hr^-1^) and downwind distance X (m); C_V = 10,000, X _= concentration predicted for 10,000 vehicles hr^-1^; E_V _and E_V = 10,000 _are the segment emission rates for volume V and 10,000 vehicles hr^-1^; and estimated parameters are k_9 _= 0.99898; k_10 _= -0.000026515; k_11 _=10749; k_12 _= -0.027061; and k_13 _= 5.2826. The portion of eq. 9 within brackets predicts the amount of enhanced or diminished dispersion relative to that occurring with V = 10,000 vehicles hr^-1^, and its value ranges from 0.89 at high vehicle flows and longer distances, to 1.29 at low vehicle flows. This is somewhat smaller than the range predicted by CALINE4, 0.88 to 1.42, since eq. 9 does not depend on wind angle. Still, this submodel performed reasonably well with an average absolute relative error of 3.4%. To reduce errors, parameters in eq. 9 could be made a function of wind angle, but this complexity did not seem warranted, especially since the largest errors (8 - 12%) occurred at low traffic volumes (< 2,000 vehicles hr^-1^), which would normally yield small concentrations.

### Composite model and model evaluation

Using eqs. 6, 8 and 9, a multiplicative model was assembled which contained 13 parameters and four input variables (wind direction, wind speed, receptor distance from roadway and traffic flow). Putting together the multiplicative components, the concentration C_X, U, V _(ppm) at distance X, wind speed U, and traffic volume V is given by:(10)

where E is the emission rate (g km^-1 ^vehicle^-1^). This can be simplified slightly(11)

where k_1_* = k_1 _k_7 _and k_4_* = k_4 _k_7_, and where parameters k_1_*, k_2_, k_3_, k_4_*, k_5_, k_6 _depend on the wind angle. Estimated parameters are shown in Table 1. With these parameters, eq. 11 predicts CO emissions (in ppm) for roads at grade, receptor distances from 15 to 300 m from the road, any wind angle, and traffic from 1,000 to 15,000 vehicles hr^-1^. (To predict concentrations in μg m^-3^, parameters k_1_* and k_2_* are multiplied by 1322.)

Figure 4 provides a visual assessment of the performance of the reduced-form model as compared to CALINE4. The overall performance was very good, e.g., the average absolute relative error was only 4.1% and there were only a few cases where errors exceed 15% (Figure 4D). The largest percentage errors (15 - 30%) were observed at distances under 30 m from the freeway and wind speeds above > 8 m s^-1^. Some of this can be seen in Figure 4A where the reduced-form model slightly over-predicted concentrations at receptors very close to the road (< 30 m) produced by wind angles of 90 and 100°C (winds parallel or nearly parallel to the road). However, for all other conditions, the reduced-form model neatly handled wind angle (Figure 4A), wind speed (Figure 4B), and traffic volume (Figure 4C).

### Evaluation at Allen Park

**Figure 5:**
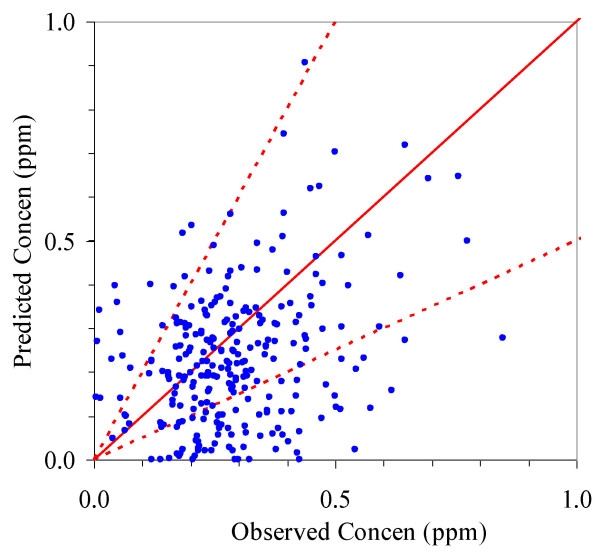
**Scatterplot of predicted versus observed 24-hour CO concentrations at the Allen Park monitoring site in 2004**. Solid line shows 1:1 line; dashed lines show factor of two boundary.

Figure 5 shows predicted and observed 24-hr CO concentrations at the Allen Park monitoring site. Considering the annual average, the model under-predicted the average measured concentration (0.33 ppm) by 33%. Considering 24-hr concentrations, 57% of predictions fell within a factor of two of observations, and the correlation coefficient was 0.33 between observed and predicted concentrations (n = 263). Model performance improved somewhat by restricting the analysis to April through October (thus omitting the colder months when CO emissions are more variable, e.g., strongly dependent on engine temperature). During this period, the average under-prediction was 25%, 59% of the 24-hr data was within a factor of two, and the correlation coefficient was 0.39 (n = 134).

Both MOBILE6.2 and CALINE4 have had previous and extensive analyses in much better controlled settings that typically show better performance. For example, Benson [11] describes three freeway studies, including those using tracer gases that largely eliminated uncertainties in the emission term, which gave 75% or more of CALINE4 predictions within a factor of two, a criterion sometimes used to define acceptable performance. Predictions of PM_2.5 _involve more complexity and larger uncertainties, especially regarding emission factors [22], thus agreement will not be as good. Only a few studies, which have been limited in extent, have evaluated roadway models for PM_2.5_, and thus little quantitative performance data are available. Tests at a relatively flat suburban site gave reasonable performance; performance deteriorated at a more complex urban site with possible street canyon effects, due to tall buildings, e.g., only 56% of predictions fell within the factor-of-two envelope of the observations [23]. Additionally, some systematic biases have been noted in CALINE4 and other roadway models, e.g., a tendency to overpredict concentrations when on-road emissions are low, and to underpredict when on-road emissions are high [24].

The "fair" or "middling" performance of the model at Allen Park can be explained by several factors. First, while we had hourly traffic counts, we used regional estimates of the vehicle mix and age distribution, and we did not account for highly emitting vehicles. This leads to considerable uncertainty in emission factors. Using vehicle mix estimates derived from the weight categories in the PTR data did not substantially alter results, but this also reduced the sample size since much of this data were unavailable. Second, we did not account for the "background" level of CO. While small, this would explain some of the under-prediction. Third, in reviewing the hourly data, it was apparent that concentrations in the early morning (midnight to 6 am) and sometimes during the morning rush hour were considerably under-predicted. This may be explained by background levels of CO, higher emissions when vehicles are cold, nocturnal inversions, and stable atmospheric conditions. The hourly data also showed that afternoon rush hour concentrations were frequently over-predicted. Fourth, Gaussian plume models have a number of well-known limitations, and they do not incorporate the more recent developments in turbulence theory. As a result, for example, hourly predictions sometimes included "zero" concentrations that corresponded to some of the more elevated CO measurements at Allen Park. Last, the performance evaluation was limited in that we examined a single site with a moderate amount of missing data, and measurement noise, especially important at the low CO levels encountered, was not considered.

This evaluation does not constitute a full evaluation or validation of the model. Rather, it demonstrates the type of performance that can be expected in applications where site-specific data are limited. For CO, we anticipate that long-term levels can be predicted reasonably well, which gives a degree of confidence in both emission and dispersion sub-models. Short-term predictions will be more uncertain. Performance for other pollutants may be much more uncertain, particularly for PM_2.5 _due to limitations in the emission estimates.

### Detroit case study

**Figure 6:**
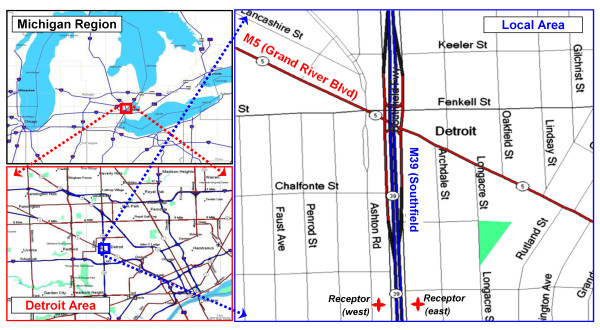
**Maps of case study area, including regional map of lower Michigan, Detroit area map, and local (modeled) area (1 km x 1.1 km) with two major roads: M39 (Southfield Expressway) and M5 (Grand River Blvd)**. UTM coordinates of local area: SW corner: (728,305 N, 208,752 E); NE corner: (729,404 N, 209,802 E). Lower part of the local area map shows two receptors (east and west of M39) used in the longitudinal analysis.

Figure 6 locates the case study area. The modeled 1 km^2 ^area is primarily residential and contains two major roads: M39 (Southfield Expressway) is a limited-access 6-lane highway oriented nearly north-south (R = 178°) with annual average daily traffic (AADT) of 144,600 vehicles day^-1^; M5 (Grand River Boulevard) is a 5-lane (including central turning lane) surface arterial street with a diagonal alignment (R = 116°) and an AADT of 30,500 vehicles day^-1 ^(SEMCOG 2005-6 traffic counts). (The receptor grid is shown in the Additional file 1: figure S1.) Additional file 1: figure S3 show hour-by-hour volume on both roads, based on midweek measurements (Tuesday through Thursdays) in 2005 and 2006 at each site (two days in each direction). Both roads show distinct rush-hour periods, although traffic volume remains relatively high from 7 am through about 8 pm. Additional file 1: figure S4 shows the vehicle mix through the day, based on typical weekday observations designed to represent freeways and arterials [19]. On freeways, the fraction of HDDV averaged 12.0% (range of 7.7-19.5%), and the HDDV and LDV fractions are negatively correlated. The highest HDDV fraction occurs early in the morning (4 - 5 am), although traffic is very light at this time (0.51% of daily total). The estimated total volumes on Saturdays, Sundays and holidays decrease by 23, 32 and 26%, respectively, from weekday values; for heavy duty vehicles, the decrease is larger, 62, 73 and 49%, respectively. The diurnal patterns on these days changes from weekdays, e.g., the morning peak largely disappears and there is an increase in late night and early morning volumes (Additional file 1: figure S2). Additional file 1: figure S5 shows estimated flows for weekdays, Saturdays, Sundays and holiday periods.

**Figure 7:**
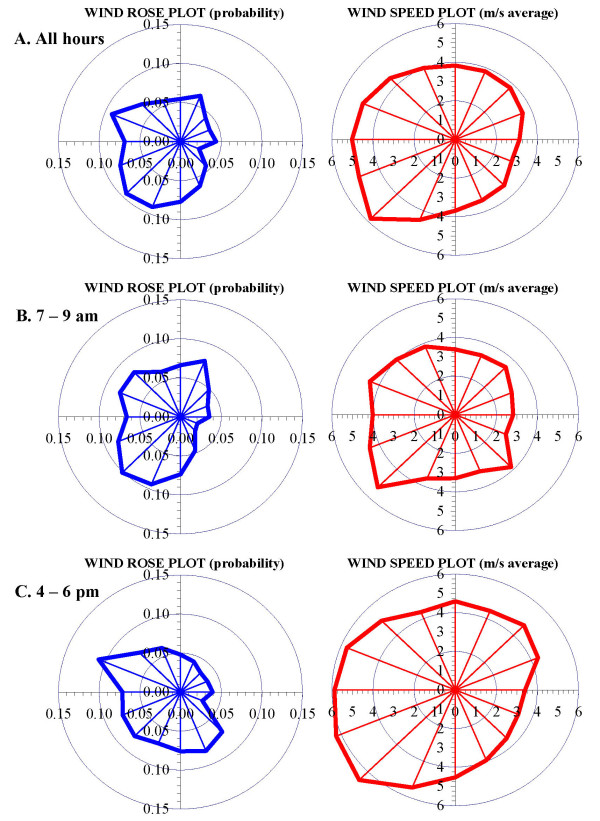
**Wind direction and speed plots for 2006 (all hours) and morning and afternoon rush hour periods**.

We briefly focus on the 2006 meteorological data. There were 688 hours of calms (when predictions were not attempted). Most days had few if any hours of calms (291 days had ≤3 hours of calms). The hourly wind speed averaged 4.29 m s^-1 ^and ranged from 1.3 to 16.5 m s^-1 ^(calms excluded). Wind direction and speed "roses" that show the probability and speed of winds in 16 sectors (each subtending 22.5°) are presented in Figure 7 for three cases: (a) all hours of the year; (b) the morning (7 - 9 am) rush hour period; and (c) the evening (4 - 6 pm) rush hour period. In the morning, moderate SW, SSW and WSW winds dominate (Figure 7b), while in the afternoon, winds shift to the WNW and are stronger; occasionally, moderate SSE and SE winds occur (Figure 7c). This diurnal variation is not represented by the annual patterns (Figure 7a). Other trends emerge when examining the lowest wind speeds that can produce the highest concentrations. As shown in Additional file 1: figures S6 - S8, which contrast winds in the morning rush hour periods on the basis of speed, the lightest winds (≤2.5 m s^-1^) arise primarily from the NNE and S sectors, directions not apparent in the annual analyses. Strong seasonal patterns are shown in Additional file 1: figures S9 - S12, e.g., winter is dominated by SW and WNW winds, spring with WNW winds, and summer with SW and light NNE. As discussed below, such seasonal and diurnal patterns can greatly influence concentration predictions.

**Figure 8:**
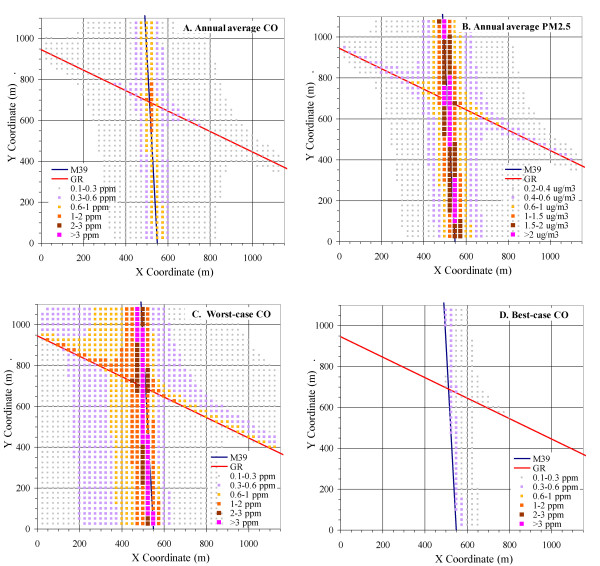
**Predicted concentrations across the case study area**. A. Annual average CO concentration. B. Annual average PM_2.5 _concentration; C. Worst-case CO day; D. Best-case CO day. M39 and GR (Grand River) are the modeled roads.

Annual average CO concentrations for the case study area show surprising symmetrical gradients about each road (Figure 8A). Across the modeled domain, CO levels averaged only 0.18 ppm (range from 0.03 to 1.15 ppm), far below the 8-hr CO standard of 9 ppm. For PM_2.5_, concentrations averaged 0.39 μg m^-3 ^(range from 0.07 to 2.4 μg m^-3^), also well below the annual standard of 15 μg m^-3 ^(Figure 8B). Even though the same traffic volume and meteorology were used, PM_2.5 _produces a somewhat different spatial pattern than CO due to differences in the hourly volume of heavy duty trucks that emit most of the PM_2.5_, as well as differences in truck volume between the two roads. For both pollutants, concentration gradients are very steep. Compared to curbside locations, for example, concentrations decreased by about 50% for receptors 60 m from the road, 75% at 100 m, and 90% at 300 m. These distances vary somewhat, depending on road orientation and other factors.

Figures 8C and 8D show the "worst-case" and "best-case" days for 24-hr CO concentrations in 2006. The worst-case day was November 24, based on the highest predicted 24-hr concentration averaged across the modeled domain. On this Friday, the concentration averaged 0.54 ppm (range from 0.04 to 5.5 ppm at individual receptors), and winds were from the S, very light and often calm (average speed = 1.8 ± 0.6 m s^-1^, excluding 9 hours of calm). Winds fell to 1.3 m s^-1 ^during the morning rush hour period. While not a predominant pattern, similar conditions (S winds < 2.5 m s^-1^) during morning rush hour occurred for about 65 hr in 2006 (Additional file 1: figure S7). Conversely, Figure 8D shows the "best-case" day, June 17, based on the lowest average concentration. On this Sunday, CO levels averaged 0.05 ppm (range from 0.00 to 0.51 ppm), the S and W winds were brisk (average 6.8 ± 2.5 m s^-1^), and wind speeds further increased during the peak traffic period (9.2 ± 0.9 m s^-1 ^from 10 am to 7 pm). Such periods are relatively common (supplemental Figure S8). For PM_2.5_, the worst case day was also November 24, 2006, and the concentration averaged 1.08 μg m^-3 ^(range from 0.09 to 10.8 μg m^-3^); the best case day was February 5, 2006, another Sunday with brisk (7.2 ± 1.6 m s^-1^) WNW winds. In all cases, the highest concentrations occurred near the intersection of the two roads and were confined to a narrow corridor along the freeway; unlike annual averages, concentration patterns were highly asymmetrical about the roads. These examples show the interplay of meteorological and emission conditions in determining near road concentrations.

**Figure 9:**
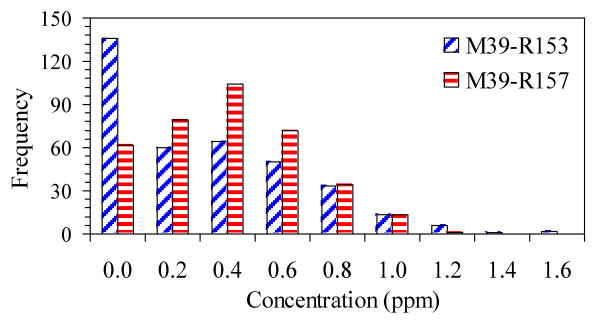
**Histogram of 24-hr CO concentration predictions for 2006 at receptors west (M39-153) and east (M39-157) of the M39 freeway**.

We examined trends of concentration predictions at various receptors, and present results for CO at two receptors located 50 m E and W of the (N-S) M-39 freeway (depicted on Figure 9). This analysis shows several important features. The first concerns the distribution of short-term concentrations. While average concentrations were higher at the E receptor (annual average = 0.39 ± 0.26 ppm) compared to the W receptor (0.33 ± 0.34 ppm), the distributions of 24-hr concentrations differed dramatically, e.g., the W receptor had many zero concentrations (resulting when the receptor was upwind of the freeway), but also the highest concentrations (Figure 9). Similar results held for the (NE-SW) arterial road. Thus, while long-term concentrations were approximately symmetrically distributed spatially, short-term concentrations at receptors on opposite sides of the road were very different, and the highest concentrations occurred on the "upwind" side due to infrequent, but low wind speeds.

**Figure 10:**
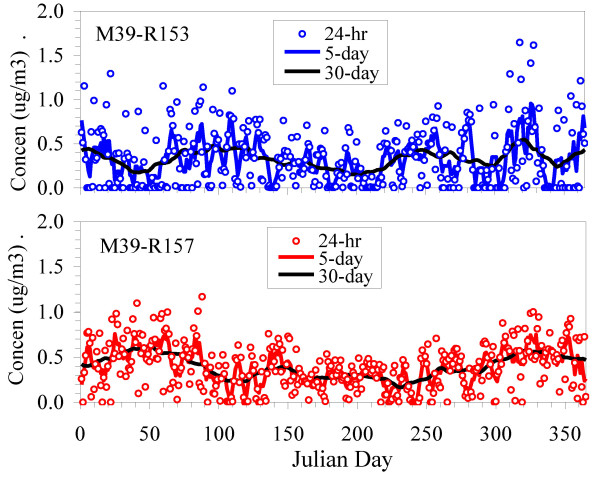
**Trends of PM_2.5 _concentrations in 2006 at two receptors west (M39-153) and east (M39-157) of the freeway**. 24-hr concentrations shown as dots; lines show 5-day, and 30-day running averages.

A second important feature concerns temporal correlation. 24-hr concentration at the two receptors on either side of the freeway (M-39) were negatively correlated, e.g., r = -0.53 for 24-hr averages and -0.28 for 5-day averages (Figure 10). Correlations were near zero (r = 0.04), however, for 30 day averages. Comparable results were found across the arterial road (M-5). In contrast, concentrations at receptors on the same side of the road were very highly and nearly perfectly correlated (r ≈ 1.0). PM_2.5 _gave similar results except that the correlations were stronger, especially at longer averaging times, e.g., r = -0.42, -0.60, and -0.67 for 24-hr, 5-day and 30-day running averages, respectively, for the M-39 receptors. This analysis shows limitations of proximity measures that do not distinguish wind direction, especially important in time series models.

A third feature is that many receptors may occasionally experience high concentrations. As examples, 98^th ^percentile and worst-case 24-hr PM_2.5 _concentrations are shown in Additional file 1: figure S13. This particular statistic is relevant for evaluating compliance with the short-term PM_2.5 _standards in the U.S. National Ambient Air Quality Standards. Across the modeled domain, the 98^th ^percentile concentrations averaged 1.38 μg m^-3 ^(range from 0.20 to 10.8 μg m^-3^), about three times the annual average, and most receptors within about 25 m of M-39 had levels above 5 μg m^-3^. Maximum concentrations were about four times the annual average. Note that the 98^th ^percentile concentrations displayed do not occur simultaneously across the modeled domain, rather, they represent the 24-hr concentration at that percentile for each receptor over the year. PM_2.5 _predictions due to roadway emissions are small relative to monitored concentrations, e.g., the annual and 98^th ^percentile 24-hr averages in 2004 at Allen Park were 15 and 37 μg m^-3^, respectively, levels similar to those measured at other Detroit area sites. The small contribution from roadways is due to the dominance of other emission sources, including long range transport of PM_2.5_, and, as discussed later, potential underestimates of PM_2.5 _emission factors from MOBILE6.2.

We give a final example of model use, motivated by LUR models intended to portray long-term average concentrations, which have often been developed on the basis of week-long monitoring campaigns. We estimate the variation expected from the long-term (annual) average, based on PM_2.5 _predictions at receptors E and W of M-39 in the Detroit case study, absolute relative error statistics, and all possible periods in 2006. For 1-week monitoring periods, the error is 43% (range from 1 - 122%) for the W receptor, and 25% (1 - 96%) for the E receptor. For 2-week periods, errors are 34% (2 - 81) and 17% (0 - 47%) for W and E receptors, respectively. LUR studies sometimes use multiple week-long measurements in different seasons to estimate annual averages. For sampling in two (opposite) seasons, the average error is lowered to 26% (2 - 73%) and 18% (0 - 60%) for W and E receptors, respectively. Comparable results are obtained at other sites and other pollutants. As might be deduced from earlier results, greater variability results at the receptor that is downwind less frequently. These results suggest that a seasonal sampling strategy is superior to one that uses a longer sampling period within a single season.

## Discussion

In this paper, we adapted two widely used emission/dispersion models, MOBILE6.2 and CALINE4, into a reduced-form model that is relatively simple and convenient to use. In part, our motivation was to use this model to estimate exposures in the context of an environmental epidemiology investigation. The approach also seems applicable for hot-spot analyses, risk assessment, GIS and urban planning applications, and traffic engineering purposes. The model uses a modest number of input variables to quickly generate predictions of ambient air pollutant concentrations that are comparable to those of the "parent" models. Such process-based simulation models enable the integration and synthesis of the major determinants of near-road ambient air pollution exposures: meteorology, vehicle emissions, and receptor location.

Two case studies were used to demonstrate the model's performance and the nature of near-road exposures, and they show several important findings that may not be well understood by exposure assessment practitioners. The highest concentrations are most likely to occur near intersections and downwind of major roads during periods of unfavorable meteorology (e.g., low wind speeds) and high emissions (e.g., weekday rush hour). In the Detroit case studies, CO and PM_2.5 _concentrations attributable to roadway emissions were low relative to air quality standards, however there are many limitations to this analysis: background levels must be added; PM_2.5 _emissions were significantly underestimated (see below); and public health impacts of roadway impacts appear large, based on the epidemiological evidence. Concentration gradients are steep, and receptors must be localized within 30 to 50 m to minimize errors. Both hour-to-hour and day-to-day variation are significant, and this variation results in unusual distributional and correlation characteristics, e.g., strong negative correlation for receptors on opposite sides of a road. We suspect that these findings can be generalized to many other locations, and they have important implications for epidemiological and other studies. Linking traffic volumes, vehicle emissions and dispersion models allows prediction of pollution concentrations at specific locations and times of interest, allowing subject-specific emission estimates.

The reduced-form model has limitations. First, we recognize that this is a simplified model that shares all the limitations of the underlying emission and dispersion models. For example, performance under very light winds or complex topography is unlikely to be accurate. Moreover, no predictions are made for calms, a limitation common to Gaussian plume models. This exclusion can result in significant errors and exposure misclassification, especially if calms are frequent, especially during rush hour periods. In Detroit, calms are relatively common is early morning, before the rush hour traffic period, but typically there are several days per year when calms extend entirely through the morning rush hour (based on examining 2004 - 2006 data), and we note that the worst-case day in 2006 included 9 hours of calms. In such cases, no morning prediction would be obtained, and the daily (24-hr) prediction could be significantly biased downwards. Imposing data completeness criteria, e.g., requiring that each 24-hr prediction have complete (or nearly complete) data, partially addresses this problem for model validation studies, but may perpetuate biases in epidemiological studies since exposure estimates for those days with potentially the highest concentrations are more likely to be omitted. Also, while MOBILE6.2 is a trip-based model considered to give reasonable predictions at a regional scale, it yields only approximate predictions for specific vehicles and road segments since it does not account for microscale features, e.g., specific vehicles, local temperatures, and acceleration/deceleration/cruise patterns.

PM_2.5 _predictions are especially uncertain. Unlike other pollutants, MOBILE6.2's estimates of PM_2.5 _emissions do not depend on temperature and speed, and recent tests indicate PM_2.5 _emission factors are underestimated by more than 2.3 times for heavy-duty diesel vehicles, and by 1.6 times for LDGVs and trucks, [9,25,26] and an empirical analysis of Detroit data shows even larger differences [27]. Recent work to improve the roadway emission inventories and utilize more sophisticated emission and dispersion models has begun, including MOVES and AERMOD that should advance simulation modeling and result in more accurate predictions [9]. In particular, MOVES has several capabilities that should improve emission estimates, e.g., with appropriate inputs it can provide estimates at national, county and, most importantly to the present application, project level scales, it incorporates substantial new emissions test data, and vehicle classification is based on Federal Highway Administration's system, which will facilitate the use of existing highway activity information. Potentially, MOVES' outputs can be incorporated into the reduced-form model. Second, for simplicity, we omitted parameters from the reduced-form model, including several that are known to be important (e.g., road height), and several that have moderate to marginal impacts (e.g., mixing height and stability category). Third, while predictions from the reduced-form model compare favorably to those from MOBILE6.2/CALINE4, this does not represent a validation of the model. Nor is our comparison of predictions to measurements at Allen Park a full evaluation, much less validation of the model. Similarly, our second case study examining the temporal and spatial patterns in Detroit does not represent results drawn from a spatially-validated model. Analyses using much more extensive, diverse, and representative data are needed for purposes of model validation. Fourth, the reduced-form model is designed for simple road geometries, specifically, straight segments. Curved roads cannot be represented, although this is unlikely to be important for larger roads. Fifth, like any model, accurate input data are needed to produce quality predictions. Finally, while the reduced-form model yields a wide range of outputs and is mechanistically-based, we do not have direct evidence indicating whether its predictions are better than much simpler measures, e.g., based on proximity or short-term wind measurements.

## Conclusions

After a thorough sensitivity analysis of the "parent" models MOBILE6.2 and CALINE4 to identify key parameters and inputs, we developed an efficient reduced-form model that simulates vehicle emissions and dispersion near roads. The application of this model in a real-world setting showed general agreement with monitored CO levels, although annual levels were under-predicted and the correlation between hourly and 24-hr predictions with measurements was only fair. These results are quite typical for uncontrolled field settings. In the second case study, we show that while the long term concentration gradients around roads were roughly symmetrical, this did not apply to short-term concentrations. This has important implications for monitoring campaigns aimed at characterizing pollutant levels near roads, for example, errors in estimating long-term concentrations from short-term measurements can be large, especially for upwind receptors. It also suggests that sampling during multiple seasons is preferable to extending the sampling period in a season, and that the variability is dependent on receptor location. Moreover, we saw strong negative correlation between short-term concentrations, e.g., 24-hr averages, for receptors on opposite sites of the road. Exposure assessments as well as LUR models can be improved by accounting for such variation

The reduced-form model facilitates a number of analyses. We anticipate applications deriving exposure estimates in epidemiological investigations examining the association between traffic-related pollutants and health effects, as well as in health risk assessments. Because it is computationally efficient, the reduced-form model might be used in Monte Carlo analyses, a way to address uncertainties in input parameters. The model also can help evaluate sampling designs intended to estimate means and other statistics, e.g., determining the appropriate number of monitoring sites or sampling periods.

## Competing interests

The authors declare that they have no competing interests.

## Authors' contributions

SB conceived of the study, provided its design and coordination, and drafted the manuscript. KZ carried out calculations involving MOBILE6.2, assisted with the Allen Park case study, and provided reviews of the text. RK carried out the initial sensitivity analyses of the CALINE4 model. All authors have read and approved the final manuscript.

## Supplementary Material

Additional file 1 **Supplemental figures and tables referred to in the text**. Click here for file

## References

[B1] Huang YL Batterman S Residence location as a measure of environmental exposure: A review of air pollution epidemiology studies J Exposure Assess Environ Epid 2000 10 1 66 85 10.1038/sj.jea.7500074 10703849

[B2] Lipfert FW Wyzga RE On exposure and response relationships for health effects associated with exposure to vehicular traffic J Expo Sci Environ Epid 2008 18 6 588 599 10.1038/jes.2008.4 18322450

[B3] Health Effects Institute Traffic-Related Air Pollution: A Critical Review of the Literature on Emissions, Exposure, and Health Effects http://pubs.healtheffects.org/view.php?id = 334 Special Report 17, January, 2010

[B4] Jerrett M Arain A Kanaroglou P A review and evaluation of intraurban air pollution exposure models J Exposure Anal Environ Epid 2005 15 2 185 204 10.1038/sj.jea.7500388 15292906

[B5] English P Neutra R Scalf R Sullivan M Waller L Zhu L Examining associations between childhood asthma and traffic flow using a geographic information system Environ Health Perspect 1999 107 9 761 767 10.2307/3434663 10464078 PMC1566466

[B6] Bellander T Berglind N Gustavsson P Using geographic information systems to assess individual historical exposure to air pollution from traffic and house heating in Stockholm Environ Health Perspect 2001 109 6 633 639 10.2307/3455039 11445519 PMC1240347

[B7] Lin M-D Lin Y-C The application of GIS to air quality analysis in Taichung City, Taiwan, ROC Environ Modelling Software 2002 17 1 11 19 10.1016/S1364-8152(01)00048-2

[B8] Jin T Fu L Application of GIS to modified models of vehicle emission dispersion Atmospheric Environment 2005 39 34 6326 6333 10.1016/j.atmosenv.2005.07.038

[B9] Cook R Isakov V Touma JS Benjey W Thurman J Kinnee E Ensley D Resolving Local-Scale Emissions for Modeling Air Quality near Roadways Air & Waste Manage Assoc 58 451 461 10.3155/1047-3289.58.3.451 18376647

[B10] Cohen J Cook R Bailey C Carr E Relationship between Motor Vehicle Emissions of Hazardous Air Pollutants, Roadway Proximity, and Ambient Concentrations in Portland, Oregon Environ Model Software 2005 20 7 12 10.1016/j.envsoft.2004.04.002

[B11] Benson PE CALINE4 -- A Dispersion Model for Predicting Air Pollutant Concentrations near Roadways, Report No. FHWA/CA/TL-84/15 1989 Office of Transportation Laboratory, California Department of Transportation, Sacramento, CA http://www.dot.ca.gov/research/researchreports/1981-1988/84-15.pdf

[B12] Hanna SR Hansen OR Dharmavaram S FLACS CFD air quality model performance evaluation with Kit Fox, MUST, Prairie Grass, and EMU observations Atmos Environ 2004 38 28 4675 4687 10.1016/j.atmosenv.2004.05.041

[B13] Ryan PH LeMasters GK A review of land-use regression models for characterizing intraurban air pollution exposure Inhal Toxicol 2007 Suppl 1 127 33 10.1080/08958370701495998 17886060 PMC2233947

[B14] World Health Organization Health effects of transport-related air pollution 2005 Copenhagen, Regional Office for Europe 125 165

[B15] Hart JE Laden F Puett RC Costenbader KH Karlson EW Exposure to traffic pollution and increased risk of rheumatoid arthritis Environ Health Perspect 2009 117 7 1065 1069 19654914 10.1289/ehp.0800503 PMC2717131

[B16] US Environmental Protection Agency User's Guide to MOBILE6.1 and MOBILE6.2 Mobile Source Emission Factor Model Office of Air and Radiation, EPA420-R-03-010 2003

[B17] Southeast Michigan Council of Governments Sensitivity Analysis of EPA's New Mobile Model Detroit, MI 2004 http://library.semcog.org/InmagicGenie/DocumentFolder/SensitivityAnalysisEPAmodel.pdf

[B18] Michigan Department of Transportation, 2006 Average daily traffic map archives http://mdotwas1.mdot.state.mi.us/public/maps_adtmaparchive (Accessed February 22, 2009)

[B19] Southeast Michigan Council of Governments Southeast Michigan On-Road Mobile Source Emissions Inventory Developed for the 2008 PM2.5 State Implementation Plan Submittal Detroit, MI 2008 http://www.michigan.gov/documents/deq/deq-aqd-air-aqe-Appendix-D-SEMI-Mobile_238072_7.pdf

[B20] Gianelli R Gillmore J Landman L Srivastava S Beardsley M Brzezinski D Dolce G Koupal J Pedelty J Shyu G Sensitivity Analysis of MOBILE6.0 US Environmental Protection Agency Report No. EPA420-R-02-035 2002 http://www.epa.gov/OMSWWW/models/mobile6/m6tech.htm

[B21] Dowling R Planning techniques to estimate speeds and service volumes for planning, National Cooperative Highway Research Planning Report 387 1997 National Res earch Council, Washington

[B22] Gramotneva G Brown R Ristovskia Z Hitchinsa J Morawska L Determination of average emission factors for vehicles on a busy road Atmos Environ 2003 37 465 474 10.1016/S1352-2310(02)00923-8

[B23] Yura EA Kear T Niemeier D Using CALINE dispersion to assess vehicular PM_2.5 _emissions Atmos Environ 2007 41 8747 8757 10.1016/j.atmosenv.2007.07.045

[B24] Chen H Bai S Eisinger D Niemeier D Claggett M Predicting near-road PM2.5 concentrations Trans Res Record 2009 2123 26 37 10.3141/2123-04

[B25] U.S. Environmental Protection Agency Analysis of Particulate Matter Emissions from Light-Duty Gasoline Vehicles in Kansas City EPA420-R-08-010, Office of Transportation and Air Quality and Office of Research and Development, Ann Arbor, MI 2008

[B26] U.S. Environmental Protection Agency Diesel Retrofit Technology: An Analysis of the Cost-Effectiveness of Reducing Particulate Matter Emissions from Heavy-Duty Diesel Engines through Retrofits EPA420-S-06-002, Office of Transportation and Air Quality 2006 http://epa.gov/otaq/diesel/documents/420r07005.pdf

[B27] Zhang K Batterman S Near-road air pollutant concentrations: A comparison of generalized additive models and CALINE4 Atmospheric Environment 2010 44 1740 1748 10.1016/j.atmosenv.2010.02.008

