# Supplementary material for: Prediction and analysis of near-road concentrations using a reduced-form emission/dispersion model
Source: Environ Health. 2010 Jun 25;9:29. doi: 10.1186/1476-069X-9-29 (PMC2914002; doi:10.1186/1476-069X-9-29)
Supplement: Additional file 1 — Supplemental figures and tables referred to in the text. [file 1476-069X-9-29-S1.DOC]

**Additional File 1 "Prediction and analysis of near-road concentrations using a reduced-form emission/dispersion model"**

Figure S1. Receptor map showing receptors on 25 m centers in a 45 x 43 receptor grid, and classification as to whether the receptor is within 300 m of road and whether east or west of the road.

Figure S2. Vehicle volumes along I75 near Allen Park over 2004 for all vehicles and for heavy-duty vehicles (HDVs), stratified by weekday, Saturday, Sunday and holiday periods.

Figure S3. Hour-by-hour weekday traffic volume on Grand River and M39 (SEMCOG 2009).

Figure S4. Vehicle mix on freeways (e.g., M39) and arterials (e.g., Grand River) over weekdays. Fractions for LDDVs (<0.4%) light duty diesel trucks (<0.3%), and motorcycles (<0.9%) are not shown. Derived from SEMCOG (2008).

Figure S5. Estimate hourly traffic volumes for each day type. Left panels show arterial road (Grand River); right panel shows freeway (M39). Figures omit trends for LDDVs (<0.4%), light duty diesel trucks (<0.3%) and motorcycles (<0.9%).

Figure S6. Wind direction and speed roses between 6 and 9 am for winds in 2006 (total 1302 hrs).

Figure S7. Wind direction and speed roses between 6 and 9 am for winds ≤2.5 m s^-1^ in 2006 (total 285 hrs).

Figure S8. Wind direction and speed roses between 6 and 9 am for winds >2.5 m s^-1^ in 2006 (total 1017 hrs).

Figure S9. Wind direction and speed roses for winter, 2006 (January through February).

Figure S10. Wind direction and speed roses for Spring, 2006 (March through May).

Figure S11. Wind direction and speed roses for Summer, 2006 (June through August).

Figure S12. Wind direction and speed roses for Fall, 2006 (September thought November).

Figure S13. 24-hr PM_2.5_ predictions for Detroit case study: A) 98^th^ percentile; B) Worst case day (November 24, 2006).

Table S1. Ratios of traffic volumes for weekdays, Saturday, Sunday and holidays compared to weekdays. Based on I75 traffic counts.

Table S2A. Vehicle mix factors in percent for weekday traffic on freeways. Adapted from SEMOCOG 2008.

Table S2B. Vehicle mix factors in percent for weekday traffic on arterials. Adapted from SEMOCOG 2008.
